# A scoping review on generative AI and large language models in mitigating medication related harm

**DOI:** 10.1038/s41746-025-01565-7

**Published:** 2025-03-28

**Authors:** Jasmine Chiat Ling Ong, Michael Hao Chen, Ning Ng, Kabilan Elangovan, Nichole Yue Ting Tan, Liyuan Jin, Qihuang Xie, Daniel Shu Wei Ting, Rosa Rodriguez-Monguio, David W. Bates, Nan Liu

**Affiliations:** 1 Division of Pharmacy, Singapore General Hospital, Singapore, Singapore; 2 Department of Pharmacy, University of California, San Francisco, CA USA; 3 Duke-NUS Medical School, Singapore, Singapore; 4 Artificial Intelligence Office, Singapore Health Services, Singapore, Singapore; 5 Singapore Eye Research Institute, Singapore National Eye Centre, Singapore, Singapore; 6 School of Pharmacy, National University of Singapore, Singapore, Singapore; 7 Byers Eye Institute, Stanford University, California, CA USA; 8 Department of Clinical Pharmacy, School of Pharmacy, University of California, San Francisco, CA USA; 9 Medication Outcomes Center, University of California, San Francisco, CA USA; 10 Harvard T.H. Chan School of Public Health, Boston, MA USA; 11 Division of General Internal Medicine, Brigham and Women’s Hospital, Boston, MA USA; 12 Centre for Quantitative Medicine, Duke-NUS Medical School, Singapore, Singapore; 13 Programme in Health Services and Systems Research, Duke-NUS Medical School, Singapore, Singapore; 14 NUS AI Institute, National University of Singapore, Singapore, Singapore

**Keywords:** Health care, Medical research

## Abstract

Medication-related harm has a significant impact on global healthcare costs and patient outcomes. Generative artificial intelligence (GenAI) and large language models (LLM) have emerged as a promising tool in mitigating risks of medication-related harm. This review evaluates the scope and effectiveness of GenAI and LLM in reducing medication-related harm. We screened 4 databases for literature published from 1st January 2012 to 15th October 2024. A total of 3988 articles were identified, and 30 met the criteria for inclusion into the final review. Generative AI and LLMs were applied in three key applications: drug-drug interaction identification and prediction, clinical decision support, and pharmacovigilance. While the performance and utility of these models varied, they generally showed promise in early identification, classification of adverse drug events, and supporting decision-making for medication management. However, no studies tested these models prospectively, suggesting a need for further investigation into integration and real-world application.

## Introduction

**Fig. 1:**
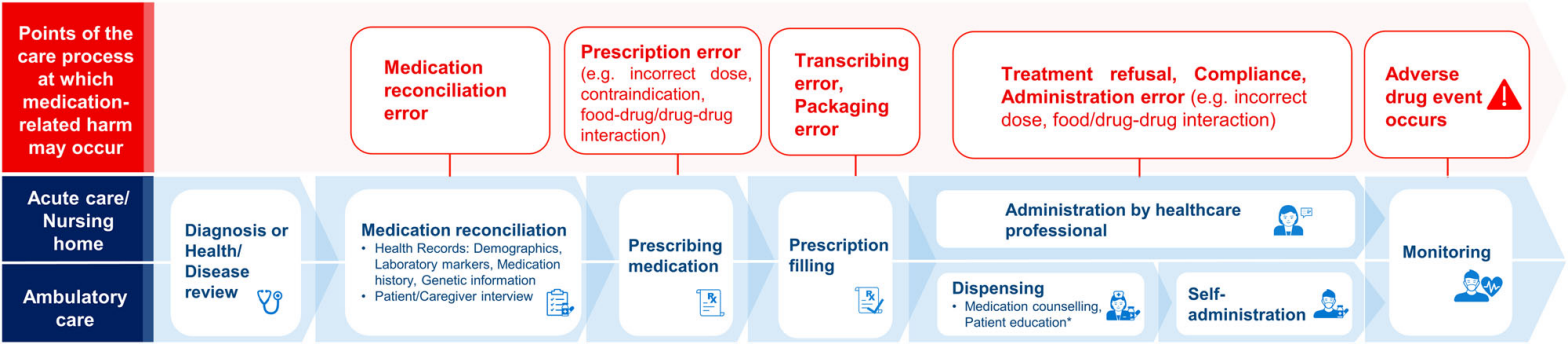
Patient care delivery and potential risks for harm. This figure shows an archetypical care delivery process and potential points of medication error. Key points in the medication use process where medication-related harm may occur spans from diagnosis to monitoring. Potential patient harm may arise from medication reconciliation errors, prescription errors, transcribing/packaging errors, administration errors, and adverse drug effects.

Medication-related harm poses significant health and economic burdens globally. In a meta-analysis conducted by the World Health Organization, the global prevalence of medication-related harm experienced by patients in medical care settings was estimated to be up to 12%. Of which, 15% was severe and fatal, resulting in a mortality rate of up 4.3 per 1000 patients^1^. In contrast, cardiovascular deaths caused by ischemic heart disease accounted for 1.09 deaths per 1000 patients^2^. The economic burden of medication-related harm is estimated at $30.1 billion and 79 billion euros in the United States and Europe respectively^3^. Medication-related harm, also referred as adverse drug events (ADEs) includes preventable or non-preventable harms caused by interventions related to medication use^4^. Preventable medication error can occur at any step from the physician prescribing medications to the patient receiving the medication. In turn, ADEs are under active surveillance during healthcare delivery to patients with health systems or pharmacovigilance activities. Figure 1 shows potential points of medication errors in the patient care delivery process.

Advances in artificial intelligence (AI), digitization of health records, and accessibility to electronic patient records have been shown to reduce the occurrence, duration, and severity of ADEs^5–7^. An AI-powered system has been reported to reduce inpatient prescribing error by up to 20%^8^. However, traditional predictive models are still limited by the lack of in-depth clinical reasoning, poor interoperability in electronic health record systems (EHRs), difficulty in detecting rare adverse events and drug-to-drug interactions. Additionally, there is a paucity of models that leverage unstructured data. In health delivery systems, overlooked serious ADEs may lead to significant healthcare complications whereas clinically insignificant ADE may be over emphasized leading to unnecessary healthcare administrative burden. Thus, generative AI (GenAI) and large language models (LLMs) may enable novel approaches that were previously unfeasible with conventional methods. For instance, preliminary studies have explored the potential of ChatGPT to recognize adverse drug reactions^9^, pharmacovigilance signal detection^10^, and automated medication chart review^11,12^.

This scoping review summarizes the breadth and depth of existing literature on how generative AI have been utilized to reduce ADEs and highlights areas for future investigation. Key concepts and definitions of terms used in this scoping review is provided in Table 1.

**Table 1:** Key concepts and definitions used in this scoping review

Concepts	Definitions
Medication related harm	The harm caused by medication if taken incorrectly, monitored insufficiently or as the result of an error, accident or communication problem^76^.
Adverse drug event	Any injury resulting from medical interventions related to a drug. This includes both adverse drug reactions in which no error occurred and complications resulting from medication errors.
Pharmacovigilance	The science and activities relating to the detection, assessment, understanding and prevention of adverse effects or any other drug-related problem.
Generative artificial intelligence	Deep learning-based AI techniques that can be used to create or produce various types of new contents, including text, images, audio, and videos, based on patterns learnt from training data. This includes foundation models and large language models.

## Results

**Fig. 2:**
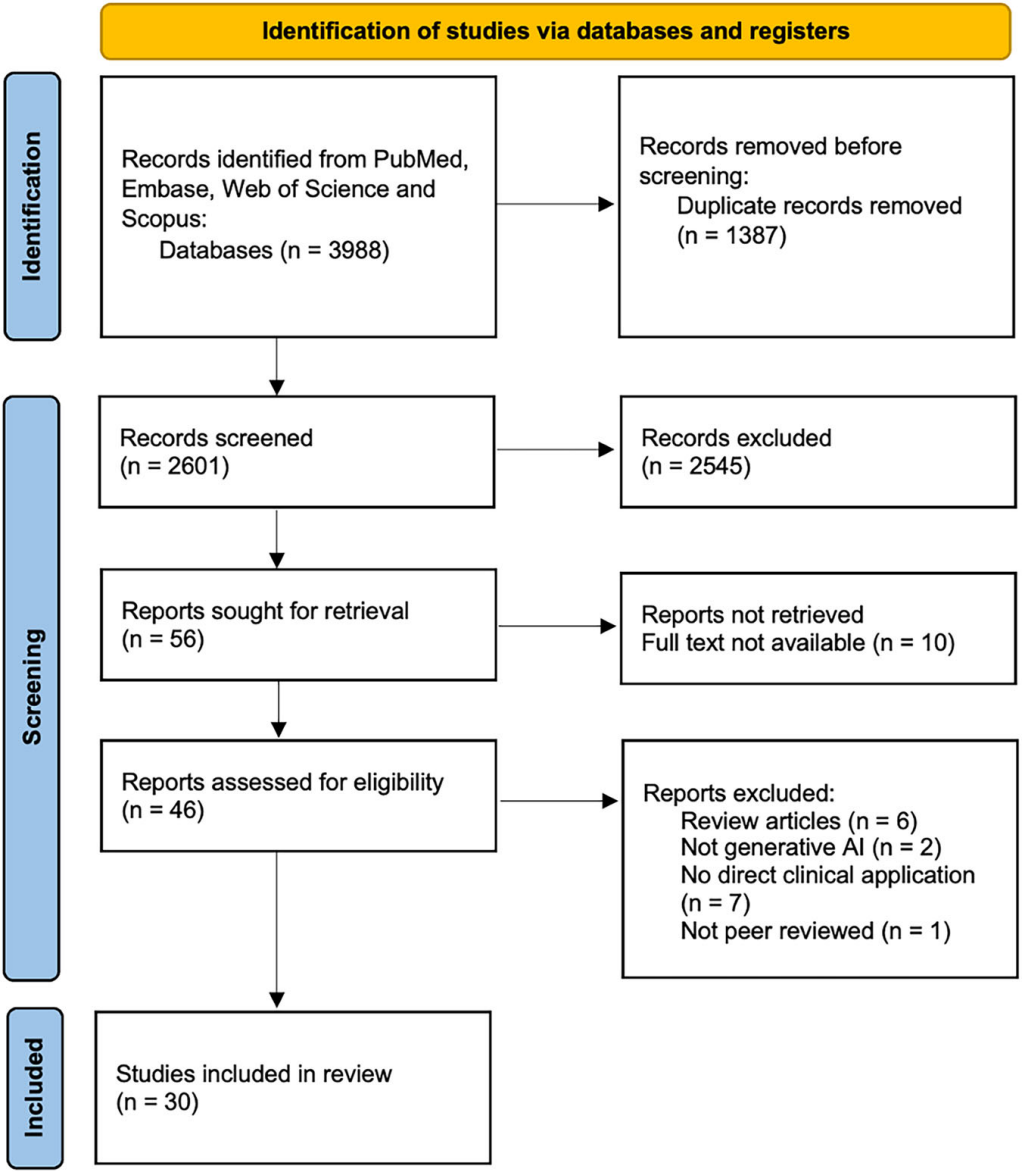
PRISMA-ScR flow diagram of paper selection. The flow diagram shows the number of records identified, included, and excluded at the different stages of the scoping review. A total of 3988 studies were retrieved from the electronic databases. After the removal of duplicates, 2601 studies were screened for inclusion and exclusion Finally, 30 studies fulfilling all inclusion criteria were included.

The search yielded 3988 articles from all databases, with 2601 remaining after removing duplicates. After applying inclusion and exclusion criteria, 30 articles were included for this review. Figure 2 shows the PRISMA-ScR flowchart of study screening, exclusion and final inclusion.

### Study characteristics

Studies evaluated the performance of GenAI models for various applications. Seven studies focused on the identification, classification, or prediction of drug-drug interactions (DDI)^13–19^. Twelve studies assessed the performance and utility of GenAI as decision support tools in medication reconciliation and deprescribing^20,21^, aid dosing calculation of crushed tablets^22^, assist medication chart review^9,23–25^, and provision of drug information^26–30^. The rest of the studies focused on the application of GenAI in adverse event monitoring from specific drug classes and enhancing pharmacovigilance processes. Study designs were predominantly observational and cross-sectional in nature. None of the studies tested models prospectively in their respective settings of application. The proposed applications of GenAI were broadly distributed across clinical (community, inpatient care) and public health settings. For the remainder of this paper, the term LLMs refers to generative LLM. Details on studies included can be found in Supplementary Table 2.

### Training, validation, and testing dataset characteristics

Pharmacovigilance tasks often used public health databases, including the FDA (Food and Drug Administration) Adverse Event Reporting System (FAERS) Public Dashboard^31^, Health Canada Adverse Reaction Online Database^32^, and China Food and Drug Adminstration^33^. Various datasets were used for named entity recognition tasks in ADE detection, such as PubMed abstracts^34^, user-generated content from web platforms (Medicitalia^35^, WebMD^36^), and open or restricted access datasets (CoNLL2003^37^, BioCreative V CDR^38^, n2c2^39^). Models built for DDI prediction utilized closed-source or in-house datasets such as DrugBank^26,40^. No model training was performed for studies that evaluate LLMs as decision support tools.

Testing datasets are often accrued from prior published studies, such as drug-drug interaction and deprescribing case scenarios^20,41^, with one study using an in-house retrospective cohort of medication prescriptions^14^. Studies that evaluate LLMs as decision support tools used case vignettes, multiple choice questions, or medication-related enquiries to test the performance of LLM outputs. These model outputs were tested against human expert-generated responses.

### Model types

Proprietary LLMs featured frequently in the reviewed studies, including various versions of ChatGPT and Google Bard. Studies used simple prompts or iterative prompting to generate responses on pre-trained LLMs. None of the studies reported the use of additional techniques to enhance model performance, such as retrieval-augmented generation or fine-tuning. One study created a tool named DELSTAR AI that utilizes the custom function of GPT-4 to provide referenceable answers from relevant peer-reviewed articles. BERT-based models (Bidirectional encoder representations from transformers) were tested in ADE identification from textual data. Two studies used a fine-tuned BERT model^34,42^ while another used a pre-trained model BioBERT using few shots learning to perform DDI identification tasks^17^.

Custom-developed models adopt iterations of generative adversarial networks (GAN) and variational autoencoders (VAE) in the studies reviewed. The GTCACS^43^ approach was a three-step approach to better identify discussion topics from social media texts. GAN achieved dimensionality reduction, keyword clustering and summarization. DeepSAVE^44^, a deep learning framework, used an enriched VAE for dimensionality reduction through parsimonious modeling of events captured on social media platform. A graph-based approach was used in a semi-Implicit graph variational auto-encoder (SIG-VAE) to predicting polypharmacy side effects through latent drug feature embeddings and graph-based predictions^18^. In one study, BART (Bidirectional and Auto-Regressive Transformers) fine-tuned with a small amount of ADE-specific named entities (few-shot learning) was adapted to allow the automated identification of diverse ADEs using small volumes of annotated data^45^. GAN was adapted in DGANDDI^16^ into a graph attention network that encode drug attributes. DGANDDI was capable of binary and multi-class prediction tasks for drug-drug interactions using an enhanced and augmented multi-dimensional dataset generated by GAN. In a similar fashion, GAN was used to generate artificial features to augment data distribution in an imbalanced spontaneous reporting dataset^33^.

### Overall model performance

For tasks that assist clinical decision making, reference standards include information from knowledge databases, e.g. Lexicomp and expert opinion (healthcare providers or pharmacologists). The most commonly reported metrics include accuracy, sensitivity, specificity, F-1 statistic, precision, recall, AUC (Area Under Curve), and AUPRC (Area Under Precision-Recall Curve). Bespoke metrics include qualitative assessment of model responses by human experts, graded on Likert scales for quality, completeness, or satisfaction. One study used ChatGPT-4 for qualitative evaluation of model performance, performed in parallel with human expert evaluation^46^.

### Performance in drug-drug interaction classification and prediction

In the prediction of potential drug interaction pairs, GAN-based models achieved high accuracy rate. GANs are generative models that learn from the distribution of data or images to create large, realistic synthetic data^47^. DGANDDI outperformed baseline methods in both binary and multi-class DDI prediction tasks. In binary prediction, it achieved an accuracy of 96.10%, AUPRC of 99.27%, and AUROC of 99.26%. In the multi-class prediction task, DGANDDI attained an accuracy of 95.89%, AUPR of 97.29%, and AUROC of 99.97%. EGFI is a BioBERT-based framework that incorporates BioGPT-2. This framework extracts DDI from 2 independent datasets with F1 scores of 0.842 and 0.720; and generates novel biomedical DDI relations not found in existing datasets^34^.

Proprietary LLMs were used to classify DDIs. One study compared the performance of different LLMs, including Microsoft Bing AI, ChatGPT-3.5 and ChatGPT-4, and Google Bard^13^. When Micromedex was used as the reference standard, the accuracy of LLMs ranged between 0.469 to 0.788, with Microsoft Bing AI demonstrating the best performance. There is currently no consensus on what is considered an acceptable performance. However, a model with an accuracy of 78.8% is unlikely to be widely adopted in clinical practice. Sensitivity was comparable across all LLMs, but specificity was significantly lower for ChatGPT-3.5 and ChatGPT-4. In another study, the Google Bard was used to screen prescriptions for drug-drug interactions, which demonstrated low degree of agreement with predictions from Lexicomp^14^. There was a nil to slight agreement between interaction risk rating (κ = 0.01), severity rating (κ = 0.02), and reliability (κ = 0.02). Conversely, ChatGPT (version not reported) was found to be highly accurate in identifying drug-drug interactions in 39 out of 40 DDI pairs tested. When prompted to explain its answer, ChatGPT produced responses that were readable and clinically reasonable.

### Performance as decision support tools

In decision support applications, a study leveraging GPT-4 for benzodiazepine deprescribing reported high degree of overall agreement between LLM and human expert in identifying cases eligible for deprescribing^20^. Agreement on four different deprescribing criteria was varied, ranging 74.7% to 91.3% (lack of indication: κ = 0.352, *P* < 001; prolonged use: κ = 0.088, *P* = 280; safety concerns: κ = 0.123, *P* = 006; incorrect dosage: κ = 0.264, *P* = 001). Qualitative analysis of GPT-4 responses found that up to 22% were ambiguous, generic and contained inconsistencies. In a separate study, GPT-3.5 was presented with a series of case vignettes involving a geriatric patient and was tasked with determining whether any medications should be deprescribed^24^. ChatGPT showed a preference for deprescribing pain medications over other drug categories, raising concerns about its potential underestimation of the importance of pain management in geriatric care. Another study introduced a web-based calculator developed to guide dosing calculation, particularly in pediatric care where such errors are prevalent^22^. The authors used ChatGPT (version not reported) and Visual Studio to write the underlying HTML code for dose division calculations and webpage interface creation. The webpage’s reliability and feasibility were then assessed using retrospective data and validated questionnaires, scoring 88.38 on the System Usability Scale. Accuracy and reproducibility of the calculator were not evaluated.

LLMs were evaluated in a broad range of clinical tasks related to prescription safety, such as medication chart review and appropriate medication dosage recommendation. In one study, ChatGPT demonstrated comparable performance to clinical pharmacists in medication counseling-related tasks^9^. Various LLM models, including GPT, Claude, and Llama, provided clinically satisfactory answers when evaluated for their capability to detect dosage errors and drug-drug interactions in prescriptions^21^. Several limitations and concerns were raised, including inaccuracies due to outdated information; unsatisfactory performance in complex tasks requiring patient-specific recommendations; and propensity to provide an answer to drug enquiries in the absence of adequate information provided in the query.

In the provision of pharmacovigilance related enquiries, ChatGPT-4 responses were compared against responses by pharmacovigilance specialists. The median score (IQR) of the ChatGPT’s responses on a 10-point Likert scale was 4.8 (3–7.3), with a specific focus on drug causality scoring lower at 3.7 (3–6.3), and information on medication and proper use scoring slightly better at 5 (3.2–8.3). The authors conclude that chatbot’s responses were generally not acceptable, especially in terms of precision and clinical relevance.

### Performance in pharmacovigilance tasks

For signal detection and ADE classification, studies used generative AI for training data augmentation and dimension reduction. These models e.g. GTCACS outperformed non-GenAI methods on internal validity measures. The DeepSAVE model, which uses a VAE approach, was tested on a dataset comprising of 104 million user search queries and 800 events. DeepSAVE outperformed existing methods (e.g., disproportionality analysis, association rule mining) with the highest F-measure across all validation datasets. A GAN-based classification model developed to automatically evaluate risk categories of drugs during post-marking surveillance demonstrated highest accuracy of 97.9% when compared against existing models.

In one study, authors demonstrated few-shot learning with LightNER and BART, and the ADE recognition performance in low-resource datasets was concluded to be acceptable. This study assessed the LightNER model’s ability to effectively transfer knowledge from a data-rich to a low-poor setting in Named Entity Recognition (NER) tasks, focusing on challenges in class and domain transfer. For instance, the LightNER model, fine-tuned using the N2C2 dataset, achieved an F1-score of 61.42%, suggesting the model’s effective transfer of task knowledge from rich-resource to low-resource settings. In a separate study, GPT-3 was employed to create an extensive dictionary of drug abuse synonyms sourced from social media. Coupled with automated API queries and simple automated filters (e.g., Google filters), the proposed method yielded a precision of 0.859 and 0.770 and recall of 0.431 and 0.395 for alprazolam and fentanyl, respectively. A BERT-based framework for ADE extraction achieved F-scores of 89.6%, 97.6%, 84.9%, and 95.9% on Twitter, PubMed, TwiMed-Twitter, and TwiMed-PubMed datasets, respectively.

LLM was harnessed to improve the efficiency of the pharmacovigilance process in one study, where authors used iterative prompting of GPT-4 to review and summarize food effects on drugs from drug review documents. Final draft summaries generated by GPT-4 were rated by FDA professionals, with 85% rated as factually consistent with reference summaries. This showcases GPT-4’s potential to aid in faster and more reliable drug assessment processes.

## Discussion

As healthcare systems increasingly prioritize patient safety, the integration of AI has the potential to enhance the detection and prevention of ADEs, and, by extension, reduce the related ADE healthcare cost. Our scoping review revealed three key applications of GenAI in the literature to date: identification and prediction of drug-drug interactions, provision of decision support in medication management, and automation of pharmacovigilance activities. This scoping review was performed to summarise the published literature on the development, validation, and testing of GenAI-based algorithms and tools designed to identify, predict or prevent medication-related ADEs. We found a wide heterogeneity in study methodologies, types of medications and disease states studied. Most articles were focused on describing the technical performance of GenAI-based algorithms while few reported validation performances. We identified key use cases and discussed their potential clinical implications, current limitations to guide future work.

Drug-drug interactions make up nearly 3% of all hospital admissions and account for up to 5% of all inpatient medication errors^48^. Harmful DDIs are often only reported from post-marketing surveillance activities, rather than at the clinical trial stage^49^. Our review included studies that predict potential DDIs pre-clinically. The performance of models augmented by GAN outperforms those trained using traditionally augmented data using a fraction of the original training dataset^50,51^. GAN can be a useful tool in enhancing prediction accuracy where data is limited. For instance, GANs leverage their capability to generate realistic synthetic data, which is particularly useful in low-resource settings like drug-drug interaction prediction. On the other hand, LLMs demonstrated variable performance in screening DDIs from prescriptions. We found that studies frequently used simple prompting strategies to elucidate responses from LLMs, with no additional techniques used to provide contextual knowledge or reduce incorrect responses (or “hallucinations”).

As decision support tools, studies adopting generative LLMs were exploratory in nature. We found a wide range of tasks and purposes (e.g., prescription review, dosage review and calculation, medication reconciliation deprescribing and answering medication inquiry). These broad applications are enabled by generalist properties of large language models, whereby a single model (e.g,. ChatGPT) is capable of performing a variety of tasks without specific pre-training^52^. LLMs demonstrate the capacity to perform tasks with little to no task-specific training, also known as “zero-shot” or “few-shots” learning. In the context of reducing medication harm, LLMs may simulate clinical reasoning and inferential skills across diverse medical disciplines, drug classes, and user settings without the need for explicit training. For instance, an LLM trained to screen prescriptions for inappropriate benzodiazepine use may be adapted easily to screen for inappropriate drug use in geriatric patients. In addition, LLMs are well poised as medical chatbots, given their text generation capabilities demonstrating a high degree of fluency, empathy, and personalization, even outperforming clinicians^53^. These explorations, however, also highlight existing challenges to the clinical adoption of LLMs. While studies to date are in the research phase, and no exploration in terms of auto-piloting or co-piloting as modes of clinical integration. Studies frequently report inaccurate and outdated responses from LLMs. The lack of reliability and consistency of responses using general-purpose LLMs such as ChatGPT precludes its autonomous use in clinical settings. Systematic bias was observed in one study, where LLMs preferentially deprescribed pain medications as compared to other medication categories in a geriatric case vignette. This was a concerning finding given the disregard for adequate pain management in geriatric patients. LLMs are susceptible to cognitive biases, for example, confirmation bias and frequency bias^54^. In addition, LLMs may perpetuate biases pervasive in healthcare, such as gender and racial bias, exacerbating health inequities^55–57^. There is a lack of standardized approach to evaluation, monitoring framework and effective risk mitigation strategies to this challenge.

A promising area of GenAI application in enhancing efficiency and impact is in ADE monitoring and pharmacovigilance, where GenAI tools may enhance the timeliness and accuracy of ADE detection from specific medication classes. Our review has shown that both transformer-based and GAN-based models enhanced the accuracy of signal detection, disease and drug entity recognition over conventional natural language processing tools. In most studies, GenAI LLMs were able to handle a wide breadth of data sources (i.e., electronic health records, online databases, and social media platform), facilitating the detection of rare events and offering a generalist capability that is essential for continuous learning and adaptation^58^. GenAI LLM-based screening of content in online medical forums, social media, or published articles showed promise for rapid identification of real-world signals. This was demonstrated in one study to screen for thyroid medication related ADE in content generated by online users of a medical forum. This automated detection of ADE was capable of providing a fine characterization of patients along different dimensions, such as co-morbidities, symptoms, and emotional state. Automation of specific tasks in pharmacovigilance that is traditionally resource-intensive is a potential avenue for productivity gain with the use of GenAI LLM models^59^.

In our review, we are unable to provide conclusive evidence that GenAI will reduce medication-related harms when applied in clinical settings. Along the continuum of medication use process, GenAI models have been adopted for specific clinical domains and tasks. Generative AI applications to inform clinical decision-making focused on specific clinical domains and tasks with the potential to be applied in medication safety. Non-generative AI models were evaluated in reducing ADEs were evaluated in a prior study^60^. A variety of AI techniques were described including neural networks and tree-based algorithms in predicting potential ADEs and enhancing early detection. Utilizing diverse data sources like genetic information and electronic health records, these AI models aimed to inform clinical decisions on safe prescribing and medication management.

A large majority of studies reviewed focused exclusively on reporting model performance on retrospective test datasets except in one study, whereby ChatGPT was compared against registered pharmacist in a parallel arm study design^9^. In this study, clinical questions based on real-world prescriptions were presented to both ChatGPT and clinical pharmacists. Despite the parallel arm study design, the primary outcome measure, i.e., the ability to answer viva-style questions, is not a standard or clinically accepted measure of the efficacy of a medical intervention or device. While the performance of ChatGPT was shown to be comparable with clinical pharmacists in drug counseling, clinical pharmacists outperformed ChatGPT in all other domains, such as prescription review and ADE recognition. Current evidence from this review suggests that state-of-the-art models are not primed for autonomous deployment in medication safety related tasks that involves clinical reasoning and judgment. In another study, providing physicians with access to an LLM as a diagnostic tool did not lead to a significant improvement in clinical reasoning compared to traditional resources. However, the LLM on its own outperformed both physician groups, highlighting the necessity for advancements in technology and workforce training to fully harness the potential of physician-AI collaboration in clinical settings^61^.

A critical evaluation of studies included in our review revealed a lack of adherence to reporting guidelines for AI studies. We did not perform a quality review of the studies in view of the scoping nature of this review and diverse hypotheses of the studies included. Checklists and reporting guidelines such as the MI-CLAIM for transparent model reporting^62^, TRIPOD + AI checklist for comprehensive reporting of predictive models^63^ and DECIDE-AI checklist for early-stage clinical evaluation of AI-based decision support tools^64^ could be adopted in future studies. However, there is still a lack of validated reporting tools for LLM-based AI model, though initial efforts have been made to create LLM-specific frameworks^65^. Evaluation or discussions on model fairness, bias, and other ethical considerations such as data privacy were also found to be lacking in the included studies.

Our study is limited by the heterogeneity of applications across reviewed publications. Varied types of GenAI tools were applied to different settings, which prevented a formal assessment of predictive validity. The diversity of training and testing datasets used precludes the generalizability of findings across different demographic groups. Patient outcomes were not reported in all studies, limiting any quantitative analyses. Furthermore, the observational nature of our study limits our ability to establish the direct impact of GenAI tools on patient outcomes when implemented in real-world settings. We limited our review to only peer reviewed articles. We acknowledge that the field of generative AI and LLM is rapidly evolving, and a large number of studies may still be in the preprint stage or archived.

We described various GenAI LLM-based methods applied to medication safety applications in this review. We found that different technologies excel in distinct areas depending on their design and application. Domain-specific language models such as BioBERT and BioGPT-2 perform well in extracting biomedical relationships due to their specialized pretraining. In contrast, LLMs demonstrate contextual reasoning and human-like response generation. However, we observe limitations with suboptimal sensitivity, specificity, and the lack of real-time data. Hallucinations remain an unresolved challenge. These require additional mechanisms such as retrieval augmented generation (RAG) or fine-tuning to allow LLMs to tailor responses to specified tasks through the provision of contextual knowledge (e.g., drug-drug interaction database)^66–68^. The advantage of such techniques has been shown in other clinical tasks, including differential diagnosis, evidenced-based decision support, and patient chart review^69,70^. These techniques, however, may rely on well-curated, clinically adjudicated clinical datasets that are not often freely available.

Instead of applying generative AI models in tasks that mandate deterministic outputs, we propose that LLMs can be adopted in ways to reduce cognitive workload for healthcare professionals. Healthcare professionals work with high volumes of multi-modal patient data and are required to pay close attention to details, synthesize information, and make clinical decisions in real time. High cognitive load poses risk for burnout and medical errors^71^. For example, LLMs can be used to analyse and reduce alert burden in electronic medical records, in medication incident analysis, and summarization in a similar fashion to discharge notes generation^12,72^. In a study published after we completed the literature search, LLMs were used in a co-pilot system to extract key named entities of online submitted prescriptions and assemble them into coherent instructions^73^. This system was shown to reduce near-miss events and improve the efficiency of pharmacy operations in a large-scale online pharmacy. Finally, LLMs can be leveraged upon as a tool in patient education and engagement thereby enhancing patient access to critical medication-related information^74^.

Future research may focus on developing and benchmarking generative AI models against established healthcare standards to further validate their performance and cost-effectiveness to ensure their safe integration into clinical practice. There is a need to develop expert curated, high-quality training datasets with diverse representation from different geographical, ethnic and social groups. Such datasets, when shared, can facilitate and accelerate the training of GenAI models adapted for different applications in the medication use process. For example, an expert-annotated dataset of incident reports can be used to fine-tune an LLM-based model to predict risk for medication incidents. Other areas of high interest include the use of LLMs for real-time monitoring of drug safety and the exploration of GAN the synthetic generation of training data, which can help overcome the limitations posed by rare ADE occurrences.

In conclusion, GenAI and LLMs have shown potential in enhancing medication safety and reducing medication-related harm. Peer-reviewed studies highlight promising areas for future implementation. However, the current applications address only a subset of the critical issues in medication safety today. Moreover, these studies often lack research rigor and comprehensive ethical evaluation. Future research should focus on addressing gaps, such as the absence of high-quality datasets specifically addressing medication safety tasks. Given the rapidly evolving nature of this field, continuous updates of this review may be needed.

## Methods

### Search strategy and selection criteria

This scoping review was conducted according to the PRISMA for Scoping Reviews (PRISMA-ScR) guidelines^75^. We searched PubMed, Web of Science, Embase, and Scopus to identify studies published between 1^st^ January 2012 to 15^th^ October 2024, related to the application of generative AI in reducing medication-related harm. Our detailed search strategy can be found in Supplementary Table 1.

Studies were included if they were published in English, described the development or application of generative AI in mitigating potential medication-related harms in the care delivery process and were peer-reviewed original research, review and viewpoints, or structured reviews of the literature reported in accordance with PRISMA-ScR guidelines. We excluded conference abstracts, case reports, studies that utilized solely predictive modeling approaches or investigated ADEs related to dietary supplements or use of medication not prescribed for the individual.

Based on these criteria, abstracts were screened for eligibility by two independent reviewers using the Rayyan online tool^76^. If no exclusion criteria were apparent in the abstract, it was included for manuscript review. Full-text manuscripts reviews were conducted by two independent reviewers. Studies that did not meet the selection criteria were excluded at this stage. In cases of discrepancy between reviewers, eligibility was determined by a third reviewer.

### Data analysis

We used an Excel sheet to extract pertinent information, including study characteristics, model details, application setting, outcome measures, findings, and reported challenges and limitations. We did not perform a critical appraisal of study quality as our primary objective is to characterise the scope of research in this field, identify research trends and gaps. The wide range of study designs and outcomes also precluded the application of a uniform quality assessment criterion.

## Supplementary information

Supplementary Information

## Data Availability

Data extracted from included studies and data used for all analyses are provided in Supplementary Table 2.

## Code availability

There are no codes developed in this study.
