# Supplementary material for: A scoping review on generative AI and large language models in mitigating medication related harm
Source: NPJ Digit Med. 2025 Mar 28;8:182. doi: 10.1038/s41746-025-01565-7 (PMC11953325; doi:10.1038/s41746-025-01565-7)
Supplement: Supplementary file 1 — Supplementary Information [file 41746_2025_1565_MOESM1_ESM.pdf]

Supplementary Table 1: Detailed search strategy

	Advanced Search Query Syntax			
**Component**	**PubMed**	**Web of Science**	**Embase**	**Scopus**
**Generative AI and Large Language Models**	("Generative AI "[tiab]) OR("Generative artificial intelligence "[tiab]) OR ("Large language"[tiab]) OR ("GPT-* "[tiab]) OR ("Variational Autoencoder"[tiab]) OR ("generative adversarial"[tiab]) OR ("GAN "[tiab]) OR ("GANs"[tiab]) OR ("bard "[tiab]) OR ("stable diffusion "[tiab]) OR ("midjourney "[tiab]) OR ("dall-e "[tiab]) OR ("Variational Autoencoders"[tiab]) OR ("Variational Auto-encoder"[tiab]) OR ("Variational Auto-encoders"[tiab]) OR ("Variational Auto encoder"[tiab]) OR ("Variational Auto encoders"[tiab]) OR ("BART"[tiab]) OR ("GLaM"[tiab]) OR ("LaMDA"[tiab]) OR ("PaLM"[tiab]) OR (“Gemini*[tiab] OR (“Mistral*[tiab] OR (“Mixtral*[tiab] OR ("LLaMA"[tiab]) OR (“Med-PaLM”[tiab] OR (“Med-LLM”[tiab]) OR "transformer architecture"[tiab] OR "BERT"[tiab] OR "self-attention mechanism"[tiab]	TS=(("Generative AI ") OR ("Generative artificial intelligence") OR ("Large language Model") OR ("GPT-*") OR ("Variational Autoencoder") OR ("generative adversarial") OR ("GAN") OR ("bing") OR ("bard") OR ("stable diffusion") OR ("midjourney") OR ("dall-e") OR ("Variational Autoencoders") OR ("Variational Auto-encoder") OR ("XLNet") OR ("BART") OR ("GLaM") OR ("LaMDA") OR ("PaLM") OR ("YaLM") OR ("LLaMA") OR (“Gemini”) OR (“Mistral”) OR (“Mixtral”) OR ("LLaMA") OR (“Med-PaLM”) OR (“Med-LLM”) OR ("transformer architecture") OR ("BERT") OR ("self-attention mechanism")	('generative ai':ab OR 'generative artificial intelligence':ab OR 'large language':ab OR 'gpt':ab OR 'generative adversarial':ab OR 'gan':ab OR 'gans':ab OR 'bard':ab OR 'stable diffusion':ab OR 'midjourney':ab OR 'dall-e':ab OR 'variational autoencoder':ab OR 'variational auto-encoder':ab OR 'language model':ab OR 'machine learning':ab OR 'deep learning':ab OR 'artificial intelligence':ab OR 'xlnet':ab OR 'bart':ab OR 'glam':ab OR 'lamda':ab OR 'palm':ab OR 'yalm':ab OR 'llama':ab OR “gemini” OR “mistral” OR “mixtral” OR “LLaMa” OR “med-PaLM” OR “Med-LLM” OR "transformer architecture" OR "BERT" OR "self-attention mechanism")	TITLE-ABS-KEY("Generative AI "OR "Generative artificial intelligence "OR "Large language"OR "GPT-* "OR "Variational Autoencoder” OR "generative adversarial" OR "GAN "OR "GANs" OR "bard "OR "stable diffusion "OR "midjourney "OR "dall-e "OR "Variational Autoencoders "OR "Variational Auto-encoder "OR "Variational Auto-encoders "OR "Variational Auto encoder "OR "Variational Auto encoders" OR "XLNet" OR "BART"OR "GLaM" OR "LaMDA" OR "PaLM" OR "YaLM" OR "LLaMA" OR “Gemini” OR “Mistral” OR “Mixtral” OR "LLaMA" OR “Med-PaLM” OR “Med-LLM” OR "transformer architecture" OR "BERT" OR "self-attention mechanism")
	AND	AND	AND	AND
**Adverse Drug Reactions (ADRs)**	"Pharmacovigilence"[Mesh] OR "Incident reporting [tiab]" OR "Adverse Drug Reaction Reporting Systems"[Mesh] OR "pharmacovigilance" [tiab] OR "Drug toxicit*"[tiab] OR "Side effect"[tiab] OR "Adverse drug event*"[tiab] OR "Adverse drug reaction*"[tiab] OR "Drug-Related Side Effects and Adverse Reactions"[Mesh] OR "Overdose*"[tiab] OR "Drug overdose"[MeSH] OR "Contraindication*"[tiab] OR "Poison*"[tiab] OR "Drug Eruptions"[Mesh] OR "Drug Hypersensitivity"[Mesh] OR "Drug Hypersensitivity Syndrome"[Mesh] OR "drug reaction*"[tiab] OR "adverse drug*"[tiab] OR "drug hypersensitivit*"[tiab]	TITLE-ABS-KEY "Pharmacovigilance" OR "Drug Incident reporting" OR "Adverse Drug Reaction Reporting" OR "Drug Side effect" OR "Adverse drug event" OR "Adverse drug reaction" OR "Contraindication" OR "Drug Eruption" OR "Drug Hypersensitivity"	TS=(("Pharmacovigilance") OR ("Drug Incident reporting") OR ("Adverse Drug Reaction Reporting") OR ("Drug Side effect") OR ("Adverse drug event") OR ("Adverse drug reaction") OR ("Contraindication") OR ("Drug Eruption") OR ("Drug Hypersensitivity"))	pharmacovigilance':ab OR 'adverse drug reaction':ab OR 'hypersensitivity':ab

**Drug Interactions**	"Drug Interactions"[Mesh] OR drug interaction*[tiab] OR pharmacokinetic interactions[tiab] OR pharmacodynamic interactions[tiab])	TITLE-ABS-KEY "Drug Interaction" OR "Pharmacokinetic interaction" OR "pharmacodynamic interactions" OR "Drug-Drug Interaction"	TS=(("Drug Interaction") OR ("Pharmacokinetic interaction") OR ("pharmacodynamic interactions") OR ("Drug-Drug Interaction"))	("drug interaction":ab)
**Medication Use Process**	"Medication Errors"[Mesh] OR medication error*[tiab] OR "Prescription Errors"[Mesh] OR prescribing errors[tiab] OR prescription errors[tiab] OR "Contraindications, Drug"[Mesh] OR "omission error[tiab]" OR "Medication Reconciliation"[Mesh] OR medication reconciliation[tiab] OR drug reconciliation[tiab] OR "Polypharmacy" [Mesh] OR "deprescribing" [Mesh] OR "deprescribe*[tiab] OR dispensing error[tiab] OR administration error[tiab] OR transcribing error[tiab] OR transcription error[tiab]	TITLE-ABS-KEY "Medication Error" OR "Prescription Errors" OR "Contraindications" OR "Medication Reconciliation" OR "Polypharmacy" OR "Deprescribing" OR "Dispensing error" OR "Administration error" OR "transcription error"	TS=(("Medication Error") OR ("Prescription Error") OR ("Prescribing Error") OR ("Contraindications") OR ("Medication Reconciliation") OR ("Polypharmacy") OR ("Deprescribing") OR ("Dispensing error") OR ("Administration error") OR ("transcription error"))	("Medication error":ab) OR ("deprescription":ab) OR "(transcription error":ab)

**Patient Compliance / Adherence**	"Medication adherence"[Mesh] OR adherence[tiab] OR "Patient compliance"[Mesh] OR Medication persistence[tiab] OR drug persistence[tiab] OR drug adherence[tiab] OR drug compliance[tiab] OR "treatment refusal"[Mesh] OR remote monitoring[tiab] OR remote sensing[tiab]	TITLE-ABS-KEY "Medication adherence" OR "Medication compliance" OR Medication persistence"	TS=(("Medication adherence") OR ("Medication compliance") OR ("Medication persistence"))	("medication compliance":ab)
**Patient / HCP Education**	"Patient education as topic"[Mesh] OR medication counselling[tiab] OR drug counselling[tiab] OR "health education"[Mesh] OR patient interview[tiab]	TITLE-ABS-KEY "Patient education" OR "Medication counselling" OR "Drug counselling" OR "health education" OR "patient interview"	TS=(("Patient education") OR ("Medication counselling") OR ("Drug counselling") OR ("health education") OR ("patient interview"))	(("patient education") AND (("drug therapy") OR "prescription"))

Supplementary Table 2: Details of studies included in review. Studies are described according to intended applications e.g. DDI identification or decision support. Studies are mostly observational in nature, evaluating either a custom-build model or a proprietary LLM. Data sources used for training and validation were diverse.

SN	Author	Year	Category	Study Design	Training and Validation Dataset	Description of Specific Task	GenAI Model and Comparator(s)	Key Results
1	Théophile Tiffe et al.	2024	ADE Identification	Model Testing	ADE Corpus V2: A dataset of PubMed abstracts annotated for drugs and adverse drug events (ADEs)	Extract drugs and their associated ADEs from unstructured text (Named Entity Recognition)	GPT-3.5-turbo (OpenAI) vs BiLSTM with self-attention, GRU with embedding-level attention and Fine-tuned BERT (state-of-the-art)	GPT-3.5-turbo (Few-shot Learning): Precision: 83.6%, Recall: 88.7%, F1 Score: 86.0%
2	Jordan Guillot et al.	2024	ADE Identification	Model Testing	4183 clinical progress notes within 30 days of any CAR-T administration from 253 patients extracted from University of California, San Francisco (UCSF) deidentified clinical data warehouse.	Extract ADEs related to CAR-T administration from clinical notes	GPT-4 (accessed through a HIPAA compliant Microsoft Azure Studio API) to extract ADEs resulting in clinical intervention and BERTopic for topic modeling (to cluster all ADEs to identify trends over time)	GPT4 was able to extract CAR-T related adverse events with 64% accuracy
3	Hussain, S et al.	2021	ADE Identification	Model Development and Validation	Twitter Dataset, PubMed Dataset	Identify ADEs for Pharmacovigilance	FARM (FastAI + Transformers + Scikit-learn) for fine-tuning BERT models.	F1 Scores: Twitter Dataset: 0.76, PubMed Dataset: 0.82
4	Anita Elaine Weidmann et al.	2024	ADE Identification	Model Development and Validation	39 internationally recognized scientific publication databases, pharmacovigilance databases, and product characteristic databases.	Identify ADEs for Pharmacovigilance	Customized Large Language Model (LLM) named DELSTAR, based on GPT-4.	No quantitative results reported
5	Giorgio Grani et al.	2021	ADE Identification	Model development, cross-sectional	7,942 discussion threads related to Thyroid and 42,058 unrelated discussion threads (84.1%) on Medicitalia, a medical Italian website forum in which users interact with each other, and a section dedicated to online medical consultations.	Identify Thyroid related ADEs for Pharmacovigilance	GTCACS: A novel approach using a variation of GAN, agglomerative hierarchical clustering, and summarisation of the most relevant words of the clusters.	GTCACS achieved the best performance scores based on all the selected metrics. This automated detection of ADR was capable of providing a fine characterization of patients along different dimensions, such as co-morbidities, symptoms, and emotional states.
6	Faizan Ahmad et al.	2022	ADE Identification	Model Development and Validation	3 event databases: Verified ADE from the US Food and Drug Administration (FDA), HealthCanada, and the US National Highway Transportation Safety Agency (NHTSA)	Identify ADEs for Pharmacovigilance	Variational auto endocers (VAEs)	Fmeasure (35.2%), Precision (26.6%), Recall (52.0%), Timely (0.73%)

7	Chang, Chia-Hsuan et al.	2023	ADE Identification	Model Development and Validation	Trained and validated on 3 annotated datasets: CoNLL2003, BC5CDR and N2C2, tested on MigraineReviews dataset from WebMD reviews for Topamax, a drug for treating migraine.	Identify ADEs for Topiramate for Pharmacovigilance	BART (Bidirectional and Auto-Regressive Transformers)	Based on 5-shots: F1-score (26.22%), precision (40.28%), recall (19.93%)
8	Kristy A. Carpenter et al.	2023	ADE Identification	Model Development and Validation	DrugBank	LLMs to build a lexicon of colloquial drug synonyms that can be used for pharmacovigilance against drug abuse on social media	GPT-3 (OpenAI)	GPT-3 was repeatedly queried for synonyms of drugs of abuse and generated terms were filtered using automated Google searches and cross-references to known drug names. Best performing pipeline results as follows:<br>Alprazolam - Precision 0.698 Recall 0.859 F1 Score 0.770 F2 Score 0.821<br>Fentanyl - Precision 0.568 Recall 0.793 F1 Score 0.662 F2 Score 0.735
9	Jianxiang Wei et al.	2021	ADE Identification	Model Development and Validation	China's Food and Drug Admininstration spontaneous reporting database	GAN based pipeline for identification of ADE for pharmacovigilance	GAN for data augmentation and SMOTE as a data sampling method, minority expansion	Best performing model pipeine used a combination of feature enhancement with GAN and SMOTE, reaching accuracy of 97.90%
10	Guojun Dong et al.	2024	ADE Identification	Observational	Vaccine Adverse Event Reporting System (VAERS); data collected during 2020-2022	LLM in identifying vaccine related ADE	Traditional NLP, pubmedBERT vs GPT 3.5 (gpt-3.5-turbo-16k)	GPT-3.5 achieved an accuracy of 78% in correctly assigning Vaccine related ADE from reports
11	Lei Huang et al.	2022	DDI Identification	Model Development and Validation	DDIs 2013, DTIs dataset	Predict Drug-Drug Interactions (DDIs)	EGFI comprising of a biomedical pre-trained LLM BioGPT-2 as generator and pre-trained BioBERT as classifier.	Validation results: DDIs 2013 dataset - 0.842 (F1 Score); DTIs dataset: - 0.720 (F1 Score)
12	Fahmi Y Al-Ashwal et al.	2023	DDI Identification	Observational	(Tesing Dataset) 225 drug interaction scenarios; top 51 prescribed drugs	Identify clinically signifcant DDIs from prescriptions	ChatGPT-3.5, ChatGPT-4, Bing AI, Bard	Specificity ranged from a low of 0.372 (ChatGPT-3.5) to a high of 0.769 (Microsoft Bing AI). Microsoft Bing AI had the highest performance with an accuracy score of 0.788, with ChatGPT-3.5 having the lowest accuracy rate of 0.469.

13	Dilveen M. Sulaiman et al.	2023	DDI Identification	Observational	(Testing Dataset) 414 real-world medication prescriptions encountered at a community pharmacy	Identify clinically signifcant DDIs from prescriptions	Google Bard vs Lexi-Comp Online	Total number of DDIs identified by Lexicomp and Google Bard were 90 and 68, respectively. Cohen's Kappa (κ) values showed that there was a nil to slight agreement between Lexicomp and Google Bard regarding the DDI risk rating (κ=0.01). Regarding the severity rate, there was a slight agreement between them (κ=0.02), but in terms of reliability rate, there was no agreement (κ =-0.02).
14	Ayesha Juhi et al.	2023	DDI Identification	Observational	(Testing Dataset) 40 DDI pairs from a previous study	Identify DDIs and provide explanations	Chatgpt (OpenAI)	Accuracy 39/40. Mean Flesch reading ease score ranged between 27.64±10.85 to 29.35±10.16.
15	Zhou Yi et al.	2024	DDI Prediction	Model Development and Validation	DDIs, drug-protein interactions, and protein-protein interactions collected from SIDER, OFFSIDES, and TWOSIDES databases	Predict polypharmacy side effects	Semi-Implicit Graph Variational Auto-Encoder (SIG-VAE) with the novel SIPSE approach compared against Decagon model	AUROC: 0.946 (8.5% improvement over Decagon), AUPRC: 0.935 (12.3% improvement over Decagon)
16	Zengqian Denga et al.	2024	DDI Prediction	Model Development and Validation	DrugBank for molecular and target information. KEGG and PubChem for drug pathways and enzymes.	Predict asymmetric drug-drug interactions (ADDIs)	Variational Graph Autoencoder (VGAE), Graph Neural Networks (GNNs)	AUROC of 0.971, AUPRC of 0.964, and Accuracy of 0.913

17	Hui Yu et al.	2023	DDI Prediction	Model Development and Validation	Benchmark dataset from DrugBank	GAN based model for binary and multi-class DDI prediction tasks	Double Generative Adversarial Networks (DGANDDI) vs Deep Learning models	DGANDDI outperformed all comparator models in multi-class DDI prediction: Accuracy 0.959, AUPR 0.9729, AUROC 0.9997, Macro-F1 0.9252, Micro-F1 0.9251, Precision 0.9348, Recall 0.9256
18	Jiajing Zhu et al.	2022	DDI Prediction	Observational	Twosides constructed by Tatonetti et al.	IEEE Transactions on Pattern Analysis and Machine Intelligence	MADRL (Multi-Attribute Discriminative Representation Learning)	Improved adverse drug drug interaction prediction than baseline models such as SMLS, MLMS, SFLLN, MSLM, KGNN, and MGCN by 1.87% to 22.28%.
19	Arya Rao et al.	2024	Decision Support	Observational	(Testing Dataset) LLM was provided with 3 case viginettes obtained from a previous de-prescribing study, each consisting of a series of four sequential multiplechoice, select-all-that-apply questions	LLM in de-prescribing	GPT-3.5 (OpenAI)	ChatGpt deprescribed 2.7, 3.3 and 3.7 medications on average in vignettes with low, moderate and severe ADL impairment respectively. ChatGPT was found to be more likely to deprescribe pain medications relative to other medication types.
20	Huang, X et al.	2024	Decision Support	Parallel Arm, Interventional study	(Testing Dataset) De-identified, real-world clinical cases in a tertiary hospital in China, and the Clinical Pharmacist Competency Assessment questions	LLM in prescription review, patient medication education, ADR recognition, ADR causality assessment and drug counselling	ChatGPT (March 23 Version, OpenAI) compared with registered pharmacist	ChatGPT excelled in drug counseling (mean score: 8.77 vs. clinical pharmacists: 9.50, p=0.0791) but demonstrated weaker performance in rescription review (mean: 5.23 vs. 9.90, p=0.0089), patient medication education (mean: 6.20 vs. 9.07, p=0.0032), ADR recognition (mean: 5.07 vs. 9.70, p=0.0483), ADR causality assessment (mean: 4.03 vs. 9.73, p=0.023)
21	Kannan Sridharan et al.	2024	Decision Support	Observational	(Testing Dataset) Queries related to dosing regimen errors, drug-drug interactions, therapeutic drug monitoring, and genomics-based decision-making process	LLM in dosage recommendations (based on error identifications, therapeutic drug monitoring, genomics) and recognising DDI	GPT-3.5 (ChatGPT), Gemini Pro, Claude Instant, Llama-2-13b	For identification of dosage regimen errors, ChatGPT performed well, any errors determined to have a minor impact on patient's safety. For potential DDIs, all LLMs missed a major interaction between metoprolol and verapamil. For dosage modifications Claude-Instant provided appropriate suggestions. Major errors

								seen from Llama-2-13b. For genomics-based decision-making, Claude-Instant and Gemini offered satisfactory responses while others contained major errors
22	Iva Buzancic et al.	2023	Decision Support	Observational, cross-section	Dataset from the EuroAgeism H2020 ESR 7 Intenational study, containing data on lifestyle, nutritional status, mobility and strength, activities of daily living, frailty, cognitive status, mood, self-reported health status, healthcare utilisation, comorbidities, symptoms, presence and control of pain, falls, medication use and laboratory findings of older adults in Europe.	LLM in deprescribing benzodiazepines in elderly patients	GPT-4 (OpenAI) vs Healthcare professional	Both humans and ChatGPT identified patients for benzodiazepine deprescribing (96.1% and 89.6%, respectively), showing an agreement rate of 95% (κ = .200, p = .012). Agreement on four deprescribing criteria ranged from 74.7% to 91.3% (lack of indication: κ = .352, P < .001; prolonged use: κ = .088, p = .280; safety concerns: κ = .123, p = .006; incorrect dosage: κ = .264, p = .001). 22.1% of GPT-4 responses were ambiguous outputs, generic answers and inaccuracies, posing inappropriate decision-making risks.
23	Jehath Syed et al.	2024	Decision Support	Model and Website development	NA	LLM in designing a webpage to calculate doses of crushed tablets	ChatGPT and Microsoft Visual Basic	Dose 4 You calculator was graded as “Excellent” or Grade “A.” with a high degree of acceptability scored on a likert scale
24	Yiwen Shi et al.	2023	Food Drug Interaction	Observational	100 New Drug Applications (NDA) submitted to FDA	LLM in extracting Food-drug interaction information from NDA and generating summaries	GPT-3.5-turbo vs GPT-4	85 % of the GPT-4 generated summaries are factually consistent against reference. However, GPT-4 evaluation shows a consistency rate of only 72%.

25	Ali H. Salama	2024	Patient or Healthcare Professional Education	Observational	(Testing Dataset) 80 drug enquiries on four domains, 20 multiple-choice questions each: Drug-drug interaction, adverse drug effects, drug dosing recommendation, alternative drug therapy recommendation	LLM in handling drug enquiries	GPT-3.5 (OpenAI)	Drug-drug interactions: 6 out of 20 with success rate 30%. Adverse drug effects: 17 out of 20 with success rate 65% Drug dosing recommendation: success rate was only 35% Alternative drug therapy recommendation: high success rate with 85%
26	Yasser Albogami et al.	2024	Patient or Healthcare Professional Education	Ovbservational, cross-sectional	(Testing Dataset) 70 real-world inquiries submitted by healthcare professionals and patients, stored in the Drug Information Inquiry Database maintained by the Drug and Poisoning Information Center at the Pharmacy Administration at an academic medical city.	LLM in handling drug enquiries	GPT-3 (YouChat), GPT-3.5 (OpenAI), GPT-4.0 compared against registered pharmacists	LLMs: Accuracy ranged from 30% to 64.3%; safety (with/without provision of mitigating strategies) ranged between 80% to 95% with GPT-4 performing best in both measures. Performance of pharmacists was not reported, safety (with/without mitigation strategies) was reported to be 80%.
27	Kevin Lorenz et al.	2024	Patient or Healthcare Professional Education	Observational	(Testing Dataset) Drug enquiries in five domains of the AAAAI/ACAAI drug allergy guidelines (prevalence, symptoms, diagnostic tests, management, prevention)	LLM in handling drug enquiries related to drug allergies	GPT-3.5 (OpenAI)	Average DISCERN score of 3.44/5, indicating fair-good quality information, with 76% agreement with guideline recommendations. Fleiss k score was 0.65, which represented substantial agreement between the graders of ChatGPT responses. ChatGPT did not provide any accurate sources.
28	Elif Çoban et al.	2024	Patient or Healthcare Professional Education	Observational	(Testing Dataset) Questions prepared by an experienced oral and maxillofacial surgeon on drugs causing osteonecrosis of the jaw	LLM in handling enquiries related to osteonecrosis of the jaw	Chatgpt (OpenAI)	Average GQS score across all questions: 3.9 ± 0.8
29	Jasmin Hundal et al.	2024	Patient or Healthcare Professional Education	Observational	(Testing Dataset) Drug enquiries about 53 solid tumour drugs approved in 2020 - 2022	LLM in handling drug enquiries related to anti-tumour agents	GPT-4 (OpenAI)	**GPT-4 scored 100% correct on FDA approval & mechanism of action; 53% (28/43) on common adverse reactions; 32% (17/53) for drug warnings and precautions.** When a second search was conducted with the same question, GPT provided

								different responses in 53% (19/36), same responses in 39% (14/36).
30	François Montastruc et al.	2023	Patient or Healthcare Professional Education	Observational	Questions asked by mail or phone to Toulouse Pharmacovigilance Center	LLMs in answering drug enquiries	GPT 4.0 (OpenAI)	Median (IQR) rating of the 50 questions by Chatbot was 4.8 out of 10 (3–7.3)<br>Median (IQR) of answers regarding drug causality by Chatbot was 3.7 out of 10 (3–6.3)<br>Median (IQR) of answers regarding information on medication and proper use was 5 out of 10 (3.2–8.3)
